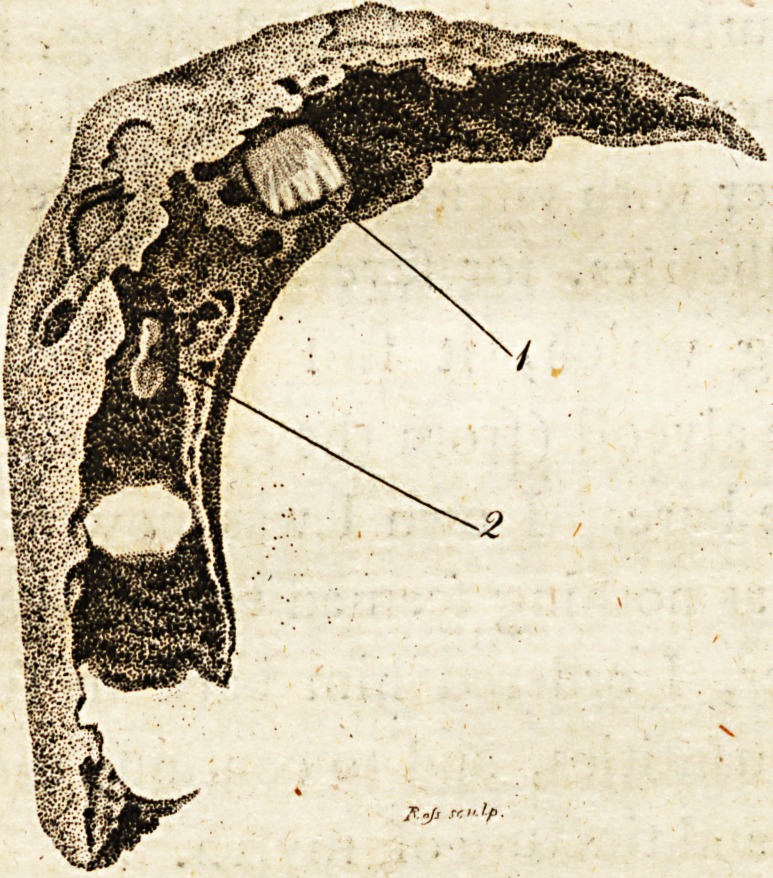# Account of a Case, in Which a Considerable Portion of the Lower Jaw Bone Was Removed; to Which Are Added Some Remarks on the Effects Produced by Matter Formed in the Socket of a Tooth, and Confined There

**Published:** 1787

**Authors:** Joseph Brandish

**Affiliations:** Member of the Corporation of Surgeons of London, and Surgeon at Alcester in Warwickshire


II. *Account of a Case, in which a considerable Portion of the lower Jaw Bone was removed; to which are added some Remarks on the Effects produced by Matter formed in the Socket of a Tooth, and confined there. By Mr.* Joseph Brandish, *Member of the Corporation of Surgeons of London, and Surgeon at Alcester in Warwickshire. Communicated in a Letter to Dr.* Johnstone, *Physician at Worcester; and by him to Dr.* Simmons.

ON the 14th of February, 1785, I was desired to see Henry Haines, of this town, a boy about five years of age. His friends told me he had had the jaundice and a bad fever, but had recovered. He complained then of a sore mouth, which they called a canker, and they had made use of several things without effect. Upon examining his mouth, I found an ulcer on the gum of the lower jaw of the left side, opposite the third of the dentes molares, which tooth was loose. His breath was very fœtid from the discharge of the ulcer, and he had some fever and colliquative night sweats. I ordered him an infusion of bark, to be taken three or four times a day, and to wash his mouth very frequently with a gargle composed of honey of roses and tincture of myrrh, and and desired them after each time of using the gargle to apply a dossil of lint, dipped in tincture of myrrh, to the ulcer.

In about a fortnight the tooth fell out; the ulcer continued spreading, and in a short time two or three more teeth became loose. The discharge was so great, that they were obliged to change the pillow he lay on three or four times a week. He was at times in great pain, which, added to the constant drivelling from his mouth, prevented his sleeping, and lowered him very much. I now touched the edges of the ulcer with vitriolic acid, lowered with syrup of mulberries, for several mornings; notwithstanding which, it still continued spreading, and the alveoli (from the gums being corroded) became bare. I own I was now at a loss what to do, as nothing seemed to be of any service: however, I ordered him bark in substance, in large quantities, and to continue the use of the gargle and tincture of myrrh.

The boy in a short time after this was much mended in his health, and in about three months a separation had evidently taken place in the jaw bone, and a small sore burst on the outside, below the condyle of the jaw, under the ear, which discharged considerably. This

I dreſſed very ſuperficially with Turner's cerate. He took the bark at this time twice or thrice a day; till being tired of it, we left it quite off. He then lived on a milk diet, and uſed the tincture of myrrh only. The bone was near five months before it became quite looſe, and I then took it out altogether, as repreſented in the plate *. The ſore on the out-

ſide immediately healed, and his mouth, by uſing the gargle before mentioned, very ſoon

* In the engraving, figure 1 refers to a tooth; and figure 2 to a tooth not yet emerged from the alveolus.

got got well, and has continued perfectly found ever fince. The boy is now quite well, and not the leaft disfigured.

This cafe, I think, clearly proves that difeafes may exift fimilar to thofe caufed by tranfplanting teeth, mentioned in Mr. Hunter's Treatife on the Venereal Difeafe, without any venereal taint, as he juftly obferves; and, in fact, without any tranfplantation of teeth at all; but, in my opinion, from matter formed in the focket of the tooth, and confined there. This, I think, will appear tolerably clearly from the following cafes: — Soon after Henry Haines got well, a child, of about the fame age, was brought to me with a fmall ulcer on the gum of the lower jaw. The tooth oppofite to the ulcer being loofe, I took it out, and thought that matter followed, but was not quite certain whether it came from the focket or the ulcer: however that may be, it had in fome degree deftroyed the bone, as it was feveral months getting well. He loft three teeth, and fome exfoliations came off from the jaw bone. He took the bark, and ufed the gargle before mentioned.

I have lately been applied to (fince I have read Mr. Hunter's cafes) to fee another child, about about four years old, who, as it was believed, had likewife a canker on the gum of the lower jaw. In this inftance alfo I found a fmall ulcer on the edge of the gum; the tooth oppofite was faft, but from the refemblance this cafe had to the other, I immediately drew the tooth, and a fmall quantity of matter iffued. I ordered the part to be wafhed with a little tincture of myrrh, and it healed without any farther affiftance.

In the cafe of tranfplanted teeth, matter feems to be formed in the focket from the irritation caufed in fixing the tooth; but the caufe of the evil in thefe children I fhall leave to the determination of others, and hope thefe hints may be of fervice to other practitioners in fimilar cafes.

*Alcefter,*

June 30th, 1787.

## Figures and Tables

**Figure f1:**